# Insights into the Genomic Background of Nine Common Chinese Medicinal Plants by Flow Cytometry and Genome Survey

**DOI:** 10.3390/plants13243536

**Published:** 2024-12-18

**Authors:** Chang An, Denglin Li, Lin Lu, Chaojia Liu, Xiaowen Xu, Shiyu Xie, Jing Wang, Ruoyu Liu, Chengzi Yang, Yuan Qin, Ping Zheng

**Affiliations:** 1 Fujian Provincial Key Laboratory of Haixia Applied Plant Systems Biology, Haixia Institute of Science and Technology, College of Life Sciences, Fujian Agriculture and Forestry University, Fuzhou 350002, China; ancher0928@163.com (C.A.); lidenglin016@163.com (D.L.); ll15966192116@163.com (L.L.); liuchaojia1069@163.com (C.L.); xuxiaowen1116@163.com (X.X.); xieshiyu927628@163.com (S.X.); wangjing051402@163.com (J.W.); liuruoyu2013@foxmail.com (R.L.); 2 Pingtan Science and Technology Research Institute, College of Marine Sciences, Fujian Agriculture and Forestry University, Fuzhou 350002, China; 3 College of Pharmacy, Fujian University of Traditional Chinese Medicine, Fuzhou 350122, China; tiebaojin@163.com

**Keywords:** herbs, genome size, flow cytometric, genome survey

## Abstract

Medicinal plants have long played a crucial role in healthcare systems, but limited genomic information on these species has impeded the integration of modern biological technologies into medicinal plant research. In this study, we selected nine common medicinal plants, each belonging to a different plant family, including *Sarcandra glabra* (Chloranthaceae), *Nekemias grossedentata* (Vitaceae), *Uraria crinita* (Fabaceae), *Gynostemma pentaphyllum* (Cucurbitaceae), *Reynoutria japonica* (Polygonaceae), *Pseudostellaria heterophylla* (Caryophyllaceae), *Morinda officinalis* (Rubiaceae), *Vitex rotundifolia* (Lamiaceae), and *Gynura formosana* (Asteraceae), to estimate their genome sizes and conduct preliminary genomic surveys. The estimated genome sizes by flow cytometry were 3.66 Gb, 0.65 Gb, 0.58 Gb, 1.02 Gb, 3.96 Gb, 2.99 Gb, 0.43 Gb, 0.78 Gb, and 7.27 Gb, respectively. The genome sizes of *M. officinalis*, *R. japonica*, and *G. pentaphyllum* have been previously reported. Comparative analyses suggest that variations in genome size may arise due to differences in measurement methods and sample sources. Therefore, employing multiple approaches to assess genome size is necessary to provide more reliable information for further genomic research. Based on the genome survey, species with considerable genome size variation or polyploidy, such as *G. pentaphyllum*, should undergo a ploidy analysis in conjunction with population genomics studies to elucidate the development of the diversified genome size. Additionally, a genome survey of *U. crinita*, a medicinal plant with a relatively small genome size (509.08 Mb) and of considerable interest in southern China, revealed a low heterozygosity rate (0.382%) and moderate repeat content (51.24%). Given the limited research costs, this species represents a suitable candidate for further genomic studies on Leguminous medicinal plants characteristic of southern China. This foundational genomic information will serve as a critical reference for the sustainable development and utilization of these medicinal plants.

## 1. Introduction

There are over 300,000 extant species of seed plants worldwide, with approximately 60% identified as having medicinal properties [1,2]. In the past ten years, there have been fundamental advancements in genome sequencing methods and strategies, and medicinal plant genomics has followed suit [3,4,5]. With the rapid development of herbal genomics, elucidating the genomes of medicinal plants has become a crucial step in advancing the related research and applications [6,7,8]. Current plant genomics research primarily focuses on agricultural crops, yielding substantial progress in decoding the genomes of staple, economic, and forage crops [9,10]. These studies have provided invaluable genomic resources for crop breeding, stress resistance improvement, and yield enhancement [11,12]. In contrast, non-crop plants, particularly medicinal and wild species, remain underexplored in genomic research. The absence of genomic information has hindered the integration of medicinal plants with modern life sciences, limiting the application of cutting-edge biological technologies in medicinal plant research [13,14]. Historically, research on medicinal plants has primarily focused on their chemical and pharmacological properties, underscoring the urgent need for studies that uncover the biological essence of these plants [15,16]. Additionally, in the work of breeding improved varieties, artificial selection can be regarded as an accelerated and targeted form of natural selection [17,18]. Without a comprehensive understanding of the evolutionary patterns of medicinal plant genomes, successful domestication and improvement are unlikely to be achieved.

Prior to genome sequencing, the determination of genome size and the acquisition of comprehensive genomic background information are essential prerequisites [19,20]. By understanding genome size, repetitive sequences, and gene content, researchers can design more targeted sequencing strategies, optimize resource allocation, and ultimately improve the quality of genome assembly [21]. Two key techniques—flow cytometry and genome survey—are employed to estimate genome size and assess genomic complexity [22,23,24]. Flow cytometry allows for the rapid and accurate estimation of nuclear DNA content, thereby enabling the calculation of genome size. Meanwhile, genome survey sequencing provides an initial analysis of the genome using high-throughput sequencing, facilitating the identification of repetitive sequences, transposable elements, and other complex features. China is one of the richest countries in traditional herbal medicine resources, with thousands of medicinal plants playing a crucial role in clinical treatment [25]. In recent years, advancements in high-throughput sequencing technologies have enabled the successful decoding of an increasing number of herbal genomes. For example, genomic studies of *Ligusticum chuanxiong* and *Lonicera japonica* have uncovered the biosynthetic pathways of their active compounds, providing a valuable foundation for breeding improvements and sustainable resource utilization [26,27]. The genome research of *Gastrodia elata* has elucidated its symbiotic relationship with fungi [28], providing critical insights for the optimization of artificial cultivation and the improvement of its quality. However, a significant number of medicinal plants with important therapeutic properties still lack fully resolved genomic information, which hinders their potential in modern herbal medicine development.

In recent years, with the development of the healthcare industry and the modernization of traditional Chinese medicine, many medicinal plants have gained attention not only for their traditional applications in Chinese medicine but also for their distinct pharmacological properties [29]. Our study focuses on nine medicinal plant species, including *G. pentaphyllum*, which is rich in saponins and is extensively applied for immune regulation, cardiovascular protection, antioxidation, and antitumor therapies [30]. *G. formosana*, a traditional medicinal and edible plant, exhibits antihypertensive, hypoglycemic, anti-inflammatory, antioxidant, and hepatoprotective properties [31]. *M. officinalis*, whose roots are used medicinally, has demonstrated efficacy in treating conditions such as sexual dysfunction, joint pain, and weakness in the lower back and knees [32]. The mild nature of *P. heterophylla* makes it suitable for long-term use in individuals with weakened constitutions, often being used as a tonic for children and the frail [33]. *R. japonica*, the primary component in various herbal formulations, is commonly employed for treating chronic liver diseases and protecting liver function [34]. *S. glabra* is widely used in the development of herbal medicines due to its anti-inflammatory and antiviral effects [35]. *V. rotundifolia* is widely applied in traditional medicine for its ability to clear heat, detoxify, dispel wind and dampness, and alleviate swelling and pain [36]. *U. crinita* exhibits significant pharmacological effects in liver protection and immune enhancement [37]. Rich in flavonoids and polyphenols, *N. grossedentata* is consumed as a health tea offering various health benefits, with dihydromyricetin exhibiting potent anti-inflammatory properties [38].

**Figure 1:**
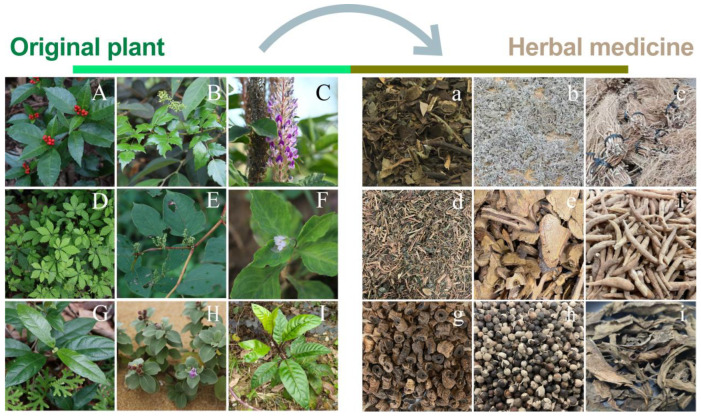
Showcasing the characteristics of 9 medicinal plants and their corresponding herbal materials. (**A**,**a**) *Sarcandra glabra*, (**B**,**b**) *Nekemias grossedentata*, (**C**,**c**) *Uraria crinita*, (**D**,**d**) *Gynostemma pentaphyllum*, (**E**,**e**) *Reynoutria japonica*, (**F**,**f**) *Pseudostellaria heterophylla*, (**G**,**g**) *Morinda officinalis*, (**H**,**h**) *Vitex rotundifolia*, (**I**,**i**) *Gynura formosana*.

In this study, we employed flow cytometry to estimate the genome sizes of the aforementioned nine medicinal plant species (Figure 1, Table S1), providing primary insights into their genomic information. Additionally, we conducted genome survey sequencing on two commonly used medicinal plants in southern China, *G. pentaphyllum* and *U. crinita*, to further assess their genome size, repeat content, heterozygosity, and GC content. These foundational data will serve as critical references for subsequent genomic investigations and offer new perspectives on the sustainable development and utilization of these medicinal plants.

## 2. Results

### 2.1. Genome Size Estimation by Flow Cytometry of Nine Medicinal Plants

**Figure 2:**
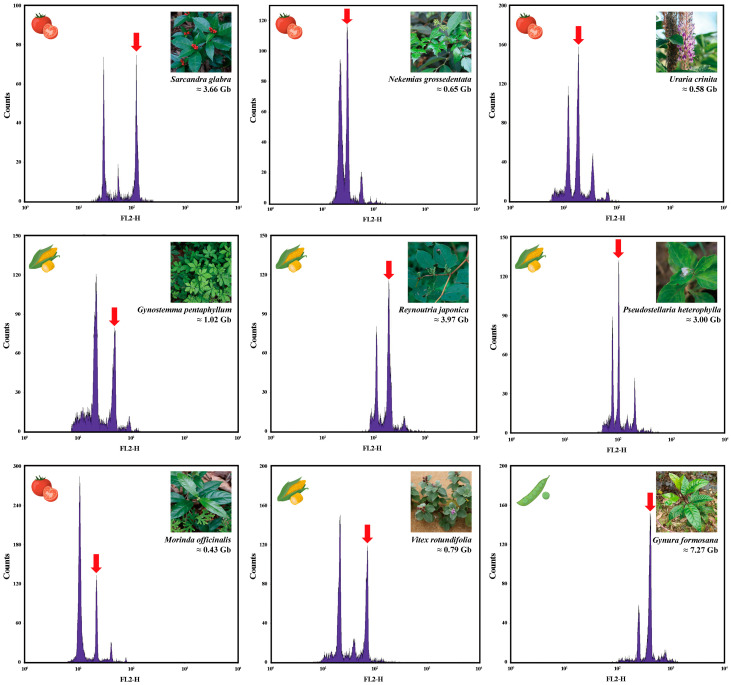
Histograms of relative PI fluorescence intensities obtained after simultaneous analysis of nuclei isolated from the internal reference species and the test plant. The horizontal axis represents fluorescence intensity, and the vertical axis represents particle number. The image in the top left corner represents the reference plant, while the arrow and the image in the top right corner depict the test species. The accompanying text describes the predicted genome size for each species.

To accurately determine the genome size of nine medicinal plants, control samples with known genome sizes are typically mixed with the test samples [39]. The fluorescence peaks of both samples are then measured under identical conditions to minimize potential errors being caused by the sensitivity of the flow cytometer. As shown in Figure 2, the flow cytometry analysis generated a high-resolution histogram, where Peak 1 represents the relative fluorescence intensity of the test sample, and Peak 2 corresponds to the fluorescence intensity of the internal standard. The genome sizes of nine medicinal plants species were estimated, based on the ratio of the fluorescence intensity between the internal standard and the test plants.

In the genome size estimation of *G. formosana*, *Pisum sativum* was used as the internal reference plant, with a reference genome size of 4.45 Gb. The fluorescence intensity of the internal reference was 249.23, while the sample’s fluorescence intensity was 407.27, resulting in an estimated genome size of approximately 7.27 Gb, which represents the largest C-value among the tested species. Additionally, *Z. mays* was selected as the internal reference for *R. japonica*, with a reference genome size of 2.3 Gb. The fluorescence intensity of the internal reference was 115.04, and the sample’s fluorescence intensity was 198.41, yielding an estimated genome size of approximately 3.96 Gb. For *S. glabra*, *Solanum lycopersicum* served as the internal reference plant, with a genome size of 0.88 Gb; the detected fluorescence intensities for the internal reference and the sample were 30.25 and 125.93, respectively, leading to an estimated genome size of 3.66 Gb. For *P. heterophylla*, with *Zea mays* as the internal reference (fluorescence intensities of 115.04 for the internal reference and 198.41 for the sample), the genome size was estimated to be 3.00 Gb. These three species exhibit genome sizes exceeding 3 Gb, indicating relatively large genomes. Lastly, the genome size estimations for *G. pentaphyllum*, *V. rotundifolia*, *N. grossedentata*, *U. crinita*, and *M. officinalis* were performed, using *Z. mays* and *S. lycopersicum* as the internal reference plants. The estimated genome sizes were 1.02 Gb, 0.78 Gb, 0.64 Gb, 0.58 Gb, and 0.43 Gb, respectively. Among these five species, only *G. pentaphyllum* exhibited a genome size exceeding 1 Gb, while the remaining four species had genome sizes below 0.8 Gb. *M. officinalis* had the smallest C-value among all the tested species (Table 1).

### 2.2. Genome Size Variation Across Corresponding Plant Families

**Figure 3:**
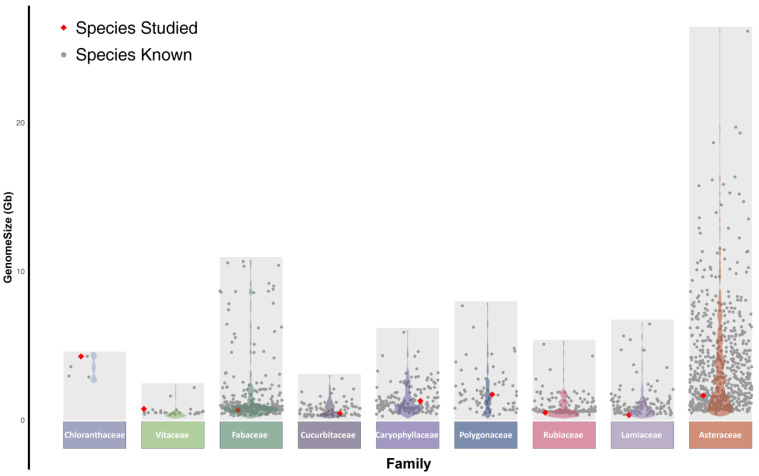
The distribution of C-values within families corresponding to the 9 medicinal plants. The studied species (represented by red diamonds) fall within the reasonable range. The violin plot shading for each family illustrates the shape of the data distribution.

To further investigate the distribution of the genome sizes of the nine plant species measured in this study within the same families, we retrieved published genome size data from related species for a comparative analysis. A scatterplot effectively visualizes the distribution of genome sizes across different plant families, highlighting the shape of the distribution, the central tendency, and the variability of the dataset (Figure 3). The results showed that genome size exhibits a broad range in multiple investigated plant families here, indicating significant variability. For instance, in the Asteraceae family, there is considerable variation in genome sizes, with *Senecio pendulus* exhibiting the largest genome (26.15 Gb) and *Conyza canadensis* the smallest (0.45 Gb), yielding a difference exceeding 25 Gb. This highlights the considerable variability within this family. Additionally, the dense distribution of genome sizes between 1 and 3 Gb suggests that Asteraceae species generally possess larger genomes, potentially indicating higher biological adaptability and evolutionary significance. Similarly, the Polygonaceae family shows a broad genome size distribution, with a long “tail”, suggesting the presence of outliers or biological subgroups (Figure 3). In contrast, species from the Vitaceae and Cucurbitaceae families exhibit a concentrated distribution in specific ranges, reflecting more consistent genome sizes. Notably, the Fabaceae family is predominantly concentrated within the range of 0.5 to 1.5 Gb, indicating lower biological variability and suggesting stability under specific environmental conditions. Furthermore, Caryophyllaceae, Cucurbitaceae, Lamiaceae, and Rubiaceae display distinct multimodal distributions, primarily concentrated in the 0.35–5.1 Gb range, indicating the possible evolution of several life forms in response to different habitats. In the Chloranthaceae family, with only four data points, all the genomes exceed 2.9 Gb, suggesting that this family typically possesses large genomes. Among the nine plants measured in this study, the genome sizes fell within the C-value range for their respective families, with the majority clustering in regions of high data density, indicating reliable measurement results.

### 2.3. Sequencing and Quality Evaluation of G. pentaphyllum and U. crinita

**Figure 4:**
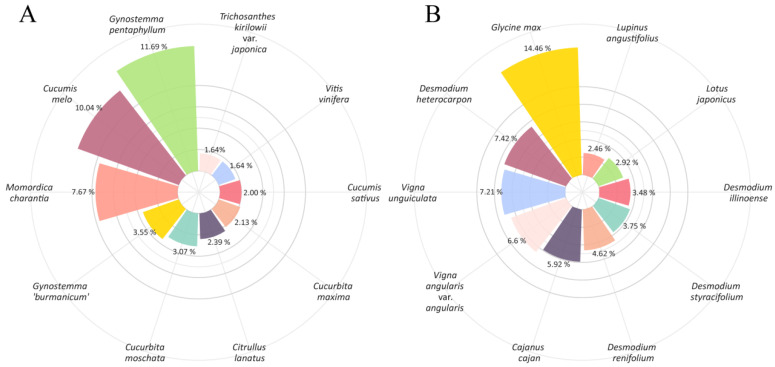
The taxonomic distribution of 100,000 quality-controlled reads, based on a BLASTn analysis against the NT Database (**A**). *G. pentaphyllum*, (**B**). *U. crinita*.

A genome survey analysis is typically employed to provide a reference for subsequent high-quality genome sequencing and assembly, as well as to evaluate the genome’s sequence ability and complexity. By combining survey sequencing with flow cytometry, a reliable estimate of the target species’ genome size can be obtained. In this study, we further selected two medicinal plants, *G. pentaphyllum* and *U. crinita*, traditionally used in southern China’s indigenous medicine [40,41], for genome survey sequencing. Using the MGISEQ-2000 sequencing platform, 53.01 Gb and 36.22 Gb of raw bases were generated, respectively, corresponding to 51.97× and 62.45× coverage, based on flow cytometry results (Table 2). The raw data were filtered using fastp, and both the raw and the clean data were subjected to quality assessments. After filtering and correction, 45.55 Gb and 33.74 Gb of clean bases were obtained, with Q20 values of 95.27% and 97.02% and Q30 values of 87.48% and 91.00%, respectively, indicating a high sequencing accuracy. Furthermore, the GC contents of the raw reads were 35.77% and 35.98%, respectively. The proportion of individual bases (A, G, C, T) is typically used to assess whether there is an AT or GC bias. The proportions of A, G, C, and T were similar, with no apparent GC bias, indicating good sequencing quality (Figure S1). Additionally, we randomly selected 100,000 quality-controlled reads from each species and used blastn to align them against the NT Database, analyzing the distribution of reads and species in the NT library. The top three matches for *G. pentaphyllum* were *G. pentaphyllum*, *Cucumis melo*, and *Momordica charantia*, all of which are members of the Cucurbitaceae family. For *U. crinita*, the top three matches were *Glycine max*, *Desmodium heterocarpon*, and *Vigna unguiculata*, all of which are members of the Fabaceae family, supporting the reliability of the sequencing data (Figure 4).

### 2.4. Genomic Characteristics Predicted by k-mer Analysis of G. pentaphyllum and U. crinita

**Figure 5:**
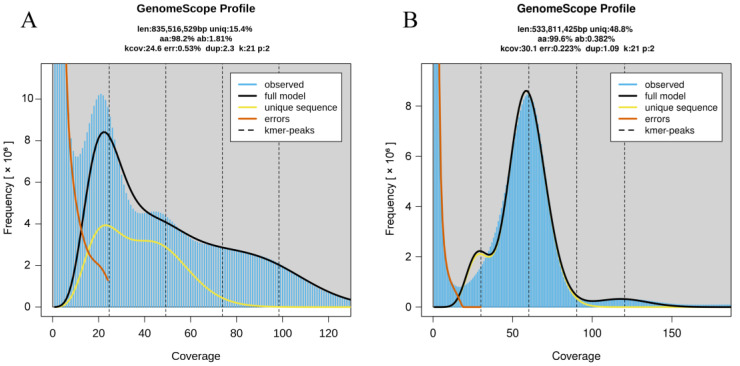
k-mer (k = 21) distribution calculated by Genomescope. (**A**). *G. pentaphyllum*, (**B**). *U. crinita*. Blue bars represent the observed k-mer distribution; the black line represents the modeled distribution without the k-mer errors (red line) and up to a maximum k-mer coverage specified in the model (yellow line). len, estimated genome length; uniq, unique portion of the genome (nonrepetitive elements); het, genome heterozygosity; err, the sequencing error rate.

A k-mer analysis was performed on the genome characteristics of *G. pentaphyllum* and *U. crinita* using the clean reads of the full sequence, with a fixed fragment length of 21-mer. In *G. pentaphyllum*, a total of 45,689,967,780 k-mers were generated, with a peak depth distribution set at 24.6×, and the genome size was estimated to be 796.81 Mb (835,516,529 bp). The estimated genome size corresponds to 76% of the genome size estimated by flow cytometry (1.02 Gb), with the heterozygosity and repeat content calculated at 1.81% and 84.64%, respectively (Table 3). Therefore, this species exhibits a highly heterozygous and complex genome. Additionally, the sequencing error rate was calculated at 0.53%. In *U. crinita*, a total of 33,642,637,212 k-mers were generated, with a peak depth distribution set at 30.1×, and the genome size was estimated to be 509.08 Mb (533,811,425 bp). The estimated genome size was similar to the genome size estimated by flow cytometry (583 Mb), with the heterozygosity and repeat content at 0.382% and 51.24%, respectively, indicating a relatively simple genome for this species. Additionally, the sequencing error rate was 0.22% (Figure 5).

## 3. Discussion

Plant genome size, as a key parameter of biodiversity [42], not only reflects differences between species and varieties but also holds significant importance in research areas such as comparative genomics, species evolution, and cell biology [43,44,45,46]. With the rapid advancement of whole-genome sequencing and genomic studies, medicinal plant genomics has opened new avenues for the study of herbal medicine [47,48]. Estimating the genome size of unsequenced medicinal plants is increasingly critical for guiding whole-genome sequencing strategies. In recent years, numerous medicinal plants, such as *Dendrobium officinale* [49], *Scutellaria baicalensis* [50], and *Ipomoea pes-caprae* [51], have undergone genome sequencing, providing complete genomic maps that offer scientific foundations for the development and utilization of genetic resources. Currently, flow cytometry has become a widely used method for genome size estimation, known for its stable results and broad applications [52]. Meanwhile, genome survey analyses not only assess genome size but also provide valuable genetic information on GC content, heterozygosity, and repeat sequences [53]. This study employed flow cytometry to determine the genome sizes of nine representative medicinal plants, while also conducting a preliminary and comprehensive genomic analysis of *G. pentaphyllum* and *U. crinita* through a genome survey, thereby enriching the DNA C-value database for medicinal plants and offering important reference data for subsequent genome sequencing and assembly efforts.

Among the nine plants measured in this study, the genome sizes fell within the C-value range for their respective families, with the majority clustering in regions of high data density, indicating reliable measurement results. The comparative analysis of genome size distribution across different families indicates distinct distribution patterns among plant families. It can be inferred that different plant families may exhibit varying adaptive evolutionary mechanisms under specific environmental conditions [54,55]. For instance, families with a broader genome size distribution, such as Asteraceae, may exhibit greater sensitivity or adaptability to environmental factors [56], whereas families with more concentrated distributions, such as Cucurbitaceae, Vitaceae, and Fabaceae, may demonstrate enhanced stability under specific conditions [57,58]. Furthermore, significant differences exist in the available genome size data across plant families. For instance, Asteraceae has accumulated 650 data points, while Chloranthaceae has only 4. This disparity is partly due to differences in the species richness across families and also reflects the current imbalance in genome size research among different families. Expanding genome size studies to include under-represented plant groups is essential for enhancing our understanding of the distribution patterns of genome sizes across families.

Among the nine species analyzed, six had their genome sizes reported for the first time, including *S. glabra* (3.66 Gb), *N. grossedentata* (0.65 Gb), *U. crinita* (0.58 Gb), *P. heterophylla* (3.00 Gb), *V. rotundifolia* (0.79 Gb), and *G. formosana* (7.27 Gb), thereby enriching the C-value data for medicinal plants. Additionally, the genome sizes of *M. officinalis* (0.43 Gb), *R. japonica* (3.97 Gb), and *G. pentaphyllum* (1.02 Gb) have been previously documented, and the comparative analysis with existing data provides insights into factors influencing plant genome size. Generally, genome size is a conserved trait for many species, serving as an intrinsic attribute [59]. Our measurement of the *M*. *officinalis* genome size was 0.43 Gb, which is comparable to the assembled genome size of the major cultivated variety “Gaoji No. 3”, assembled in 2021 [60]. As the research data accumulate, numerous studies have reported significant discrepancies in the genome size estimates for the same plant species. In our study, the genome size of *R. japonica*, as determined by flow cytometry, was 3.96 Gb, notably exceeding the 2.56 Gb reported using the k-mer analysis [61]. Different methods for genome size estimation might yield varying results. Similar findings have been reported for *Artemisia argyi* [62]. Additionally, the genome size of *G. pentaphyllum*, estimated at approximately 1.02 Gb through flow cytometry, is nearly double the previously reported range of 582 Mb to 608.95 Mb [63,64,65]. *G. pentaphyllum* is distributed across most regions of China, and studies have shown that polyploid individuals (2n = 22–88) are prevalent within this species [66]. Our sequencing samples were collected from Fujian, whereas the samples in previous studies were sourced from Guangxi and Sichuan. Polyploidy is a common adaptive response to diverse environmental conditions in plants [67], and we hypothesize that the observed polyploid characteristics in *G*. *pentaphyllum* may have evolved as an adaptive response to environmental changes, facilitating population expansion. We further employed a method called Smudgeplot, which visualizes and estimates ploidy and genome structure by analyzing heterozygous k-mer pairs. The predictive results suggest that the sequenced Gynostemma pentaphyllum is either tetraploid or pentaploid, providing additional support for our hypothesis (Figure S2). Therefore, prior to whole-genome sequencing, further analyses such as morphological observations and chromosome counting are necessary to determine the ploidy level of this species.

A genome survey can complement flow cytometry measurements, providing a more reliable understanding of genome size and offering additional insights into genome heterozygosity and repeat content, which are critical for high-quality genome assembly. Here, we also conducted genome surveys on two medicinal plants from southern China, including *G. pentaphyllum* and *U. crinita*, and found that the genome size estimated using the k-mer analysis was smaller than that obtained via flow cytometry. These results highlight that different methods for genome size estimation can yield varying results. The genome survey further revealed that *G. pentaphyllum* possesses a highly repetitive and heterozygous complex genome. A k-mer analysis, based on the frequency of short sequence fragments, often underestimates the size of highly repetitive regions [68], which are abundant in many plant genomes, such as transposable elements. Additionally, the accuracy of a k-mer analysis depends on sequencing depth. If coverage is insufficient, especially for large or complex genomes, the k-mer distribution may not accurately represent the entire genome, leading to an underestimation of genome size [69]. This issue was also observed in our study on *G. pentaphyllum*. Therefore, for species with a widespread habitat distribution or high diversity, conducting more comprehensive studies on genome size and establishing correlations between genome size and phenotypic traits or ecotypes from different regions could offer novel insights into the relationship between genome size and species adaptability.

Among the focal plant taxa, conducting genome analyses on species with simpler genomes can provide valuable reference data for related studies while also controlling research costs. Consequently, we also selected *U. crinita*, a species widely distributed in southern China with a relatively small genome, for genome survey sequencing. Genomes are generally classified as having either low heterozygosity or high heterozygosity. Additionally, genomes with a repetitive sequence content reaching or exceeding 50% are often categorized as highly repetitive genomes. Our genome survey indicated an estimated genome size for *U. crinita* of 509.08 Mb, with the heterozygosity and repeat content measured at 0.382% and 51.24%, respectively, suggesting that *U. crinita* possesses a typical diploid genome. As sequencing technologies and assembly algorithms continue to improve, most simple genomes (genome size ≤ 1 Gb, heterozygosity < 0.5%, repetitive sequence < 50%, GC content 35–65%) can be efficiently resolved through combined sequencing techniques. Recently, the combined application of HiFi and Hi-C sequencing has become a robust approach for resolving simple genomes. Using a combination of PacBio, DNBSEQ, and Hi-C sequencing, Liu et al. assembled a 762 Mb genome, with 696 Mb mapped to six pseudochromosomes, yielding a high-quality genome for *Schizonepeta tenuifolia* [70]. Li et al. employed a strategy combining PacBio and ONT long-read sequencing to obtain a 470.35 Mb genome for *Rhodomyrtus tomentosa* [71]. These methodologies provide valuable references for future genome sequencing of *U. crinita*.

## 4. Materials and Methods

### 4.1. Plant Materials

The nine medicinal plants examined in this study are widely distributed across southern China. The detailed sampling information for the specimens used in the genome size analysis is as follows: *Sarcandra glabra* (26°15′35.32″ N, 117°27′27.22″ E), *N. grossedentata* (25°02′54.08″ N, 116°32′13.98″ E), *U. crinita* (26°08′28.21″ N, 119°10′01.81″ E), *G. pentaphyllum* (24°52′34.24″ N, 117°13′50.59″ E), *R. japonica* (26°38′19.92″ N, 119°14′16.14″ E), *P. heterophylla* (27°16′51.15″ N, 119°49′40.37″ E), *M. officinalis* (24°54′21.81″ N, 117°11′51.81″ E), *V. rotundifolia* (25°37′46.21″ N, 119°46′30.81″ E), and *G. formosana* (26°4′59.41″ N, 119°14′13.05″ E). These species belong to the following families, according to the APG IV classification system for angiosperms [72]: Chloranthaceae, Vitaceae, Fabaceae, Cucurbitaceae, Polygonaceae, Caryophyllaceae, Rubiaceae, Lamiaceae, and Asteraceae. Fully developed, fresh leaf samples were immediately snap-frozen in liquid nitrogen and subsequently stored at −20 °C. For the genome size estimation of these nine medicinal plants, different internal standards were employed: *Pisum sativum* (4.45 G) for *G. formosana*; *Zea mays* B73 (2.3 G) for *R. japonica*, *P. heterophylla*, and *V. rotundifolia*; and *Solanum lycopersicum* (0.88 G) for *S. glabra*, *N. grossedentata*, and *U. crinita*.

### 4.2. Preparation of Cell Suspension and Flow Cytometry for Genome Size Estimation

The samples were placed in a Petri dish containing 0.8 mL of pre-chilled MGb dissociation solution (45 mM MgCl_2_·6H_2_O, 20 mM MOPS, 30 mM sodium citrate, 1% (*w*/*v*) PVP 40, 0.2% (*v*/*v*) Triton X-100, 10 mM Na2EDTA, 20 µL/mL β-mercaptoethanol, pH 7.5). The tissue was finely minced, perpendicular to the blade, using a sharp scalpel to form a homogenate with the dissociation solution and was allowed to sit on ice for 10 min. The plant tissue homogenate was then filtered through a 400-mesh nylon filter, resulting in a nuclear suspension. The nuclear suspension was transferred to a new centrifuge tube, and an appropriate volume of pre-chilled propidium iodide (PI) stock solution (1 mg/mL) and RNAse solution (1 mg/mL) were added [73]. The suspension was incubated on ice in the dark for 0.5–1 h, with the final working concentrations of PI and RNAse both being 50 µg/mL. The samples were analyzed using a BD FACScalibur flow cytometer (BD Biosciences, Milpitas, CA, USA). Prior to detection, the sample suspension was mixed with the internal standard suspension at an appropriate ratio to prepare the nuclear suspension sample. The flow cytometer was set to excitation at 488 nm blue light, and the fluorescence intensity emitted by the propidium iodide was recorded, collecting 10,000 particles per run. The coefficient of variation (CV%) was maintained below 5%. The data analyses and plotting were performed using Modifit 3.0 software [74].

### 4.3. Comparative Analysis of Genome Size Within Related Plant Families

To investigate the distribution characteristics of genome size within six plant families, this study employed a systematic comparative analysis approach. Genome size data for nine medicinal plant species from related families were collected from the Kew Gardens Genome Size Database and recent academic publications [75,76]. The data encompassed species from Asteraceae (650 species), Caryophyllaceae (165 species), Chloranthaceae (5 species), Cucurbitaceae (8 species), Fabaceae (277 species), Lamiaceae (131 species), Polygonaceae (75 species), Rubiaceae (126 species), and Vitaceae (34 species). These datasets were then used to conduct comparative analyses of genome sizes within each family to identify intra-family variations and potential evolutionary trends.

### 4.4. Genomic DNA Extraction and Library Construction for Sequencing

The genome survey sequencing was carried out by FutureGroup Biotechnology (Wuhan) Co., (Wuhan, China), using a whole-genome shotgun sequencing (WGS) strategy to analyze the genomes of *U. crinita* and *G. pentaphyllum*. The DNA was extracted from young leaves of both species, which were chosen for their relatively high DNA yield and quality. The DNA extraction process involved homogenizing approximately 50–100 mg of fresh tissue in liquid nitrogen and using a CTAB-based method to isolate the genomic DNA. The quality and quantity of the extracted DNA were evaluated by agarose gel electrophoresis and a Qubit Fluorometer (Thermo Fisher Scientific, Waltham, MA, USA), respectively. Only DNA with high integrity and a concentration greater than 50 ng/µL was used for the library preparation. The library preparation was performed using a standard protocol. Approximately 1–1.5 µg of genomic DNA was randomly fragmented using a Covaris sonicator (Covaris, Inc., Woburn, MA, USA) to achieve an average fragment size of 200–400 bp. The fragmented DNA was then purified using the Agencourt AMPure XP-Medium kit (Beckman Coulter Life Sciences, Indianapolis, IN, USA). The library preparation included end-repair, 3′ adenylation, adapter ligation, and PCR amplification. Following PCR, the products were purified using the AxyPrep Mag PCR Cleanup Kit (Axygen Biosciences, Union City, CA, USA). The final libraries were sequenced on the MGISEQ2000 platform (MGI Tech Co., Ltd., Shenzhen, China). To ensure the reliability of the reads, the MGI paired-end sequencing raw reads used for the genome survey were first filtered using the fastp (v.0.20.0) to remove low-quality reads, adapters, and reads containing poly-N [77]. To check for contamination, 100,000 reads were randomly selected for comparison with sequences from the NT (Nucleotide Sequence Database) library.

### 4.5. k-mer Analysis and Genome Survey

To understand the genomic characteristics, a k-mer analysis was performed using Illumina DNA data to estimate the genome size and heterozygosity prior to genome assembly. Briefly, 21-mer frequency distributions of quality-filtered reads were analyzed using the Jellyfish (v.2.3.0). By analyzing the 21-mer depth distribution of the purified sequencing reads of the 350-bp library in gce (v.1.0.2) and GenomeScope (v.2.0) software [78,79], we estimated the genome size using the following formula: G = K-num/K-depth (where K-num is the total number of 21 monomers, K-depth denotes the k-monomer depth, and G represents the genome size). The heterozygosity and repeat contents of two genomes were further estimated by combining the results of the simulated data with the different heterozygosity in *Arabidopsis* and the frequency peak distribution of the 21 k-mer analysis.

## 5. Conclusions

In this study, we employed flow cytometry to estimate the genome sizes of nine medicinal plant species from different families, including *S. glabra* (3.66 Gb), *N. grossedentata* (0.65 Gb), *U. crinita* (0.58 Gb), *G. pentaphyllum* (1.02 Gb), *R. japonica* (3.96 Gb), *P. heterophylla* (2.99 Gb), *M. officinalis* (0.43 Gb), *V. rotundifolia* (0.78 Gb), and *G. formosana* (7.27 Gb). The measured values fell within the C-value ranges for their respective families, with most clustering in regions of higher data density, indicating reliable results. Except for *M. officinalis*, *R. japonica*, and *G. pentaphyllum*, whose genome sizes have been previously reported, the genome sizes of the remaining six species are reported here for the first time, thereby significantly enriching the available data on genome sizes in medicinal plants. A comparative analysis with published data suggests that different approaches, such as flow cytometry and k-mer analyses, may yield varying genome size estimates. Even with the same method, such as a k-mer analysis, factors like genome complexity and sequencing depth can influence the results. Thus, integrating multiple methods to assess genome size can provide complementary insights and is essential for further advancing genomic research. For species with considerable genome size variation, such as *G. pentaphyllum*, population genomics studies are required to elucidate the origins, evolutionary history, and polyploid distribution patterns. Lastly, we conducted a genome survey of *U. crinita*, a medicinal plant with a small genome that is highly valued in southern China. The analysis revealed low heterozygosity and moderate repeat content, suggesting that current technologies, such as HiFi and Hi-C sequencing, can facilitate high-quality genome assembly for this species. Given the limited research costs, *U. crinita* could serve as a candidate for genomic studies on leguminous medicinal plants characteristic of southern China.

## Figures and Tables

**Table 1:** Summary of genome size estimation and fluorescence ratios in nine medicinal plants.

Species	Internal Reference Selection	Internal Reference Genome Size (Gb)	Internal Reference Fluorescence Intensity	Sample Fluorescence Intensity	Ratio	Estimated Genome Size (Gb)
*Sarcandra glabra*	*Lycopersicon esculentum*	0.88	30.25	125.93	4.16	3.66
*Nekemias grossedentata*	*Lycopersicon esculentum*	0.88	30.95	22.85	0.74	0.65
*Uraria crinita*	*Lycopersicon esculentum*	0.88	18.59	12.33	0.66	0.58
*Gynostemma pentaphyllum*	*Zea mays*	2.3	47.25	20.97	0.44	1.02
*Reynoutria japonica*	*Zea mays*	2.3	115.04	198.41	1.72	3.97
*Pseudostellaria heterophylla*	*Zea mays*	2.3	80.28	104.56	1.30	3.00
*Morinda officinalis*	*Lycopersicon esculentum*	0.88	21.86	10.75	0.49	0.43
*Vitex rotundifolia*	*Zea mays*	2.3	52.77	18.03	0.34	0.79
*Gynura formosana*	*Pisum sativum*	4.45	249.23	407.27	1.63	7.27

**Table 2:** Statistics for the sequencing data from *G. pentaphyllum* and *U. crinita*.

Sample	Total Reads	Total Bases	Clean Reads	Clean Bases	Q20 Rate (%)	Q30 Rate (%)	GC (%)
*G. pentaphyllum*	353,372,758	53,005,913,700	350,485,808	48,907,044,262	95.27	87.48	35.77
*U. crinita*	259,143,540	38,871,531,000	259,018,430	36,230,622,444	97.02	91	35.98

**Table 3:** Genomic characteristics comparison between *U. crinita* and *G. pentaphyllum*.

Property	*G. pentaphyllum*	*U. crinita*
k-mer number	45,689,967,780	33,642,637,212
k-mer cover	24.6	30.1
Homozygous (aa)	98.25%	99.64%
Heterozygous (ab)	1.81%	0.38%
Genome Haploid Length	835,516,529 bp	533,811,425 bp
Genome Repeat Length	707,171,202 bp	273,547,086 bp
Genome Unique Length	128,345,327 bp	260,264,339 bp
Model Fit	96.10%	92.26%
Read Error Rate	0.53%	0.22%

## Data Availability

The genome survey data were deposited in CNGBdb under the following accession numbers: CNP0006442 and CNP0006443. The direct link for accessing the data is https://db.cngb.org/search/project/CNP0006442/ (accessed on 15 December 2024); https://db.cngb.org/search/project/CNP0006443/ (accessed on 15 December 2024). The data supporting the findings of this study are available from the corresponding author upon reasonable request.

## Supplementary Materials

The following supporting information can be downloaded at https://www.mdpi.com/article/10.3390/plants13243536/s1. Table S1: summary of medicinal parts and pharmacological effects of nine medicinal plants; Figure S1: AT and GC content distribution of sequencing data for *G. pentaphyllum* and *U.crinita*; and Figure S2: polyploidy analysis of *G. pentaphyllum* genome, based on k-mer.
