# Supplementary figures and images for: Insights into the Genomic Background of Nine Common Chinese Medicinal Plants by Flow Cytometry and Genome Survey
Source: Plants (Basel). 2024 Dec 18;13(24):3536. doi: 10.3390/plants13243536 (PMC11679336; doi:10.3390/plants13243536)

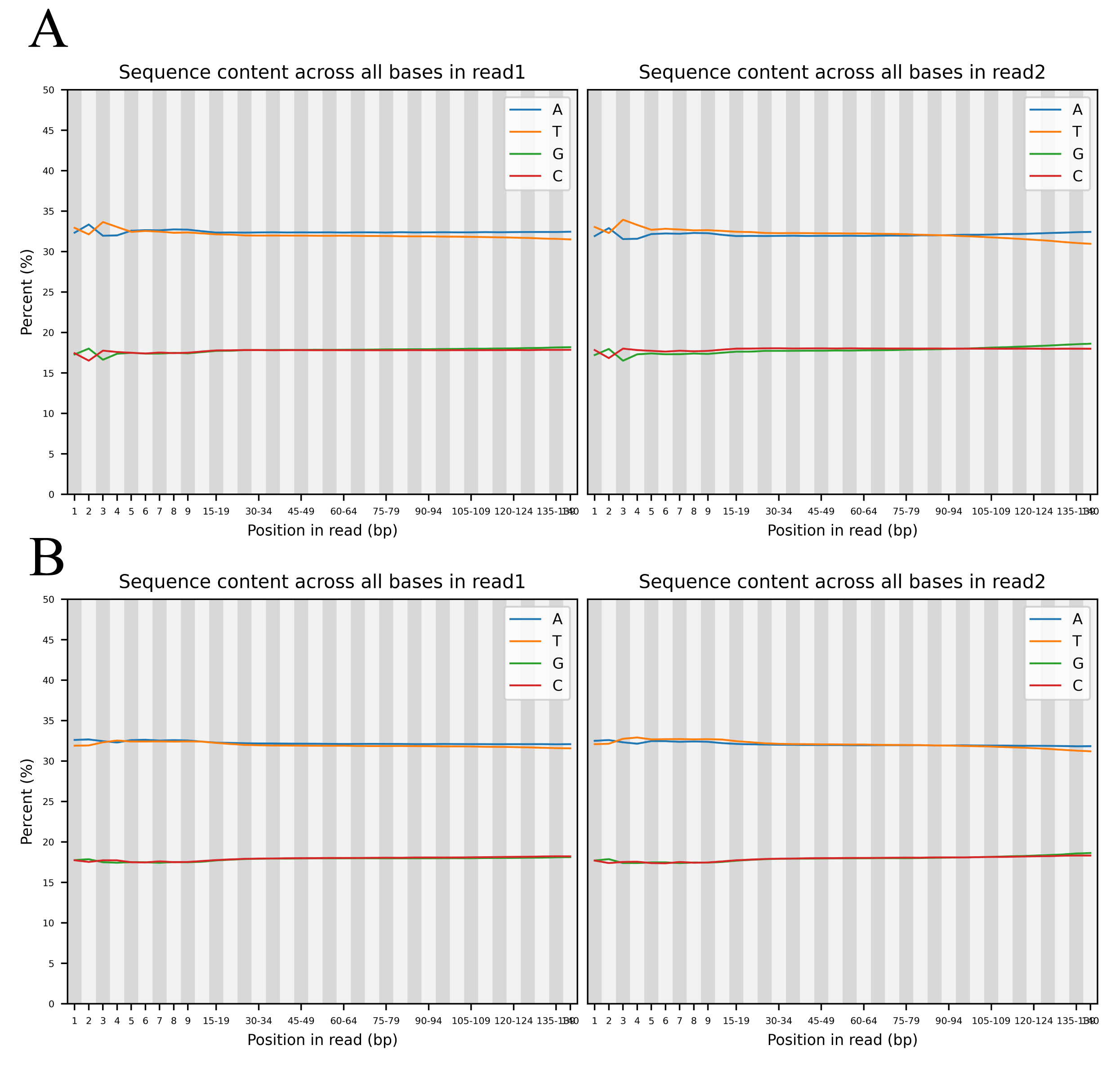

Supplement: Supplementary file 1 [file plants-13-03536-s001.zip › FigureS1.png]

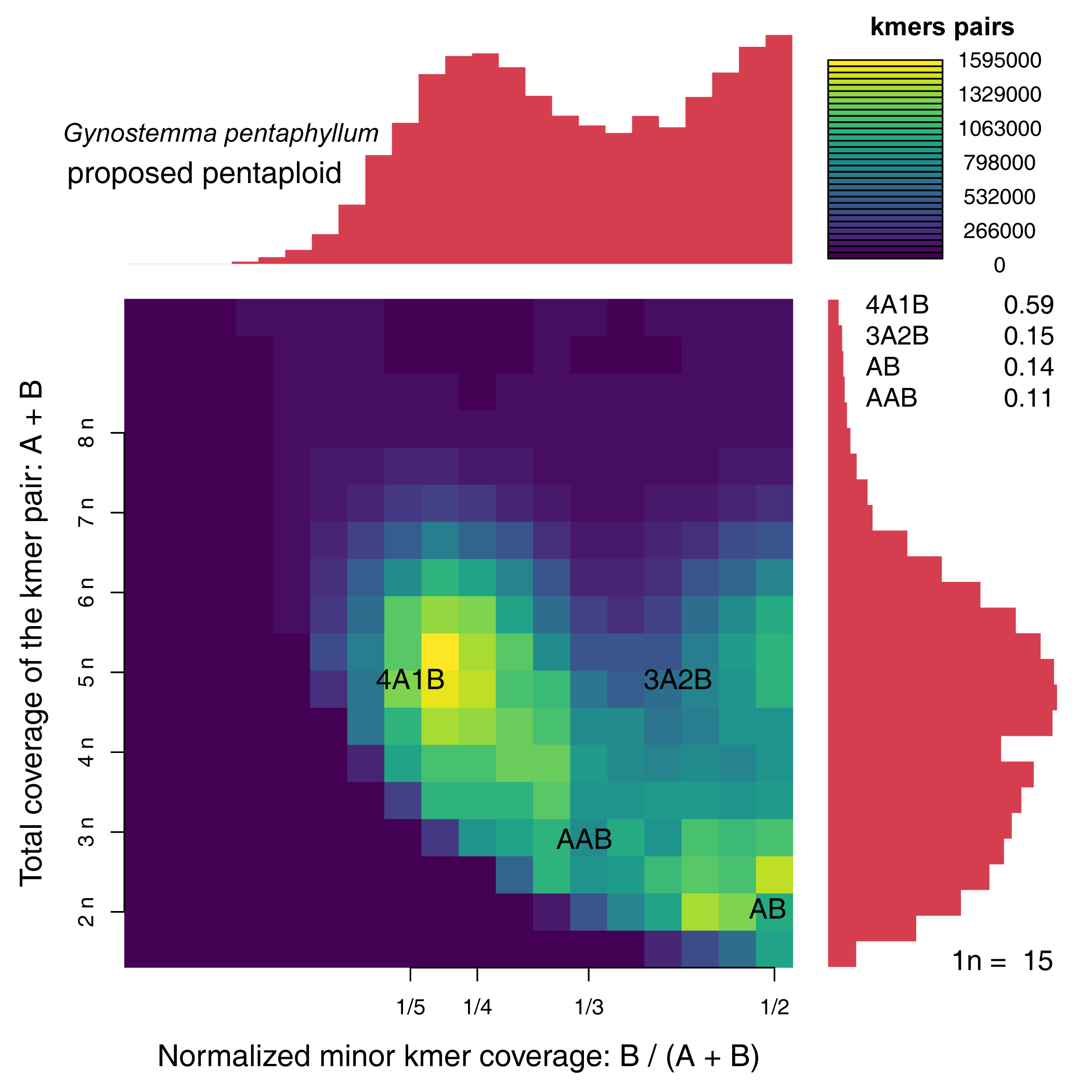

Supplement: Supplementary file 1 [file plants-13-03536-s001.zip › FigureS2.png]
